# The Optimization of Dietary Protein Level and Carbon Sources on Biofloc Nutritive Values, Bacterial Abundance, and Growth Performances of Whiteleg Shrimp (*Litopenaeus* *vannamei*) Juveniles

**DOI:** 10.3390/life12060888

**Published:** 2022-06-14

**Authors:** Abdallah Tageldein Mansour, Ola A. Ashry, Mohamed Ashour, Ahmed Saud Alsaqufi, Khaled M. A. Ramadan, Zaki Z. Sharawy

**Affiliations:** 1 Al Bilad Bank Scholarly Chair for Food Security in Saudi Arabia, The Deanship of Scientific Research, The Vice Presidency for Graduate Studies and Scientific Research, King Faisal University, P.O. Box 420, Al-Ahsa 31982, Saudi Arabia; aalsaqufi@kfu.edu.sa (A.S.A.); kramadan@kfu.edu.sa (K.M.A.R.); 2 Department of Aquaculture and Animal Production, College of Agriculture and Food Sciences, King Faisal University, P.O. Box 420, Al-Ahsa 31982, Saudi Arabia; 3 Fish and Animal Production Department, Faculty of Agriculture (Saba Basha), Alexandria University, Alexandria 21531, Egypt; 4 Faculty of Agriculture, Suez Canal University, Ismailia 41522, Egypt; oaashry707@gmail.com; 5 National Institute of Oceanography and Fisheries (NIOF), Cairo 11516, Egypt; microalgae_egypt@yahoo.com; 6 Central Laboratories, Department of Chemistry, King Faisal University, P.O. Box 420, Al-Ahsa 31982, Saudi Arabia; 7 Department of Biochemistry, Faculty of Agriculture, Ain Shams University, Cairo 11566, Egypt

**Keywords:** protein requirement, biofloc quality, crustacean farming, total heterotrophic bacteria, zero-water exchange

## Abstract

A biofloc technology-based 75-day indoor growth trial in an 80 L glass aquaria was conducted to evaluate the effects of two different carbon sources (sugarcane bagasse, SB, and wheat flour, WF) on the biofloc composition, bacterial abundance, and growth of whiteleg shrimp (*Litopenaeus vannamei*) juveniles (0.23 ± 0.04 g). Three different levels of dietary protein content (250, 300, and 350 g protein kg^−1^ diet) and two carbon sources (SB and WF) were applied (SB_250_, WF_250_, SB_300_, WF_300_, SB_350_, and WF_350_, respectively), comparing to a controlled diet without biofloc and fed on a 450 g protein kg^−1^ diet (C_450_). With the addition of SB and WF, water quality was in the ideal recommended ranges for *L. vannamei* culture. At the end of the experiment, the biofloc volume increased with increasing dietary protein levels. The nutritional value of biofloc in different treatments was influenced by dietary protein and added SB and WF. Increasing dietary protein significantly increased the protein and lipid contents of the produced biofloc. The use of WF as a carbon source significantly increased lipids and nitrogen-free extract in the biofloc. The total heterotrophic bacterial (THB) count was significantly higher (*p* < 0.05) in WF_300_ and WF_350_ than in the other treatments. The mean effect of the protein levels and carbon source was significantly reported, whereas the highest significant THB count was recorded with 300 dietary protein and using WF as a carbon source. The growth performances of *L. vannamei* fed with biofloc treatments were significantly (*p* < 0.05) higher than the C_450_ group. The highest final weight and weight gain were recorded in SB_350_ treatment. The feed conversion ratio was not affected by reducing dietary protein levels; meanwhile, the protein efficiency ratio increased significantly in biofloc treatments than in the control. Overall, the results demonstrate that, compared to the control treatment of 450 dietary protein, the biofloc treatments using WF as a carbon source could compensate for the reduction in the dietary protein levels in the diet of *L. vannamei* and maintain higher zootechnical performance.

## 1. Introduction

The shrimp aquaculture industry has recently gained considerable attention, due to aquaculture development, expansion, the high economic values of shrimp products, and the increase in human demand [1,2]. However, shrimp farming still has critical limiting factors regarding expansion, such as the cost of a shrimp diet, among other aquaculture activities [3,4]. The most important obstacle is that the diet consists of protein content (%), which has the highest feed component price [5,6]. Biofloc technology (BFT) is a realistic solution for efficiently managing water quality with low or no water exchange, enhancing shrimp growth performance and establishing an efficient and healthy shrimp culture with a better food conversion ratio in the shrimp aquaculture business [7,8,9,10,11,12]. In general, in shrimp culture, the BFT is successfully consumed, digested, and may replace a significant portion of protein in the diet [13,14,15]. BFT can be grown and produced in the culture water column and effectively reduce ammonium and nitrite accumulation in culture systems, as well as being employed as a supplemental food source for cultured shrimp [16], such as protein [17], lipid [18], amino acids [19], and fatty acids [20].

The BFT can be defined as an aggregation of microorganisms, microalgae, zooplankton, uneaten feeds, and trapped organic particles [21,22]. The BFT system needs external inputs of exogenous carbon sources to sustain the optimum carbon-to-nitrogen (C/N) ratio required for microbial production. In the BFT system, the effectiveness of BFT is determined not only by the carbon sources, but also by the ratio of carbon to nitrogen (C/N ratio) [11,23]. The goal of BFT research is to determine and describe the ideal circumstances for a diverse and stable BFT microbial community, as well as to create strategies for establishing diverse and stable BFT microbial growth [20]. The appendance of aquatic bacterial communities in ponds has a substantial impact on the internal bacterial microbiota of farmed species, which can aid in animal growth, feed consumption, water quality control, and disease resistance [24,25,26,27,28,29,30].

Several studies have evaluated the impact of using carbon sources, concentrations, and ingestions on the water quality growth performance and feed utilization of shrimp in BFT-based farms [30]. Many carbon sources were extensively investigated as sources for BFT production, such as starch, glucose, sugarcane bagasse, sugarcane molasses, rice bran, rice flour, wheat bran, wheat flour, cassava flour, gram flour, corn flour [15,22,30,31,32,33,34], as well as agriculture waste (by-products) [35,36]. Although not all carbon sources support the production of BFT with the same efficiency [15,20,30], some carbon sources are considered promising substrates for BFT aggregation due to their ability to support rapid ammonia elimination and a higher production of BFT volume [15,37]. In BFT, some carbon sources resulted in improved growth, feed utilization, water quality control, and enhanced total heterotrophic bacteria (THB) [30]. On the other hand, aquaculture wastewater contains high amounts of pollutants from unconsumed feeds and fish feces [38], resulting in high levels of total ammonia nitrogen (TAN), nitrite–nitrogen (NO_2_-N), and nitrate–nitrogen (NO_3_-N), which are considered as good applicable sources for BFT [30].

The production of whiteleg shrimp, *Litopenaeus vannamei*, in BFT-based intensive systems with zero-water exchange has become popular and has achieved sustainable growth over the last decade [12,39,40]. Several researchers have calculated the various ideal dietary protein requirements for *L. vannamei* under various farming conditions, which could be exploited to optimize growth performance, which ranged between 32–40% [14,41,42,43,44,45,46,47]. In addition, BFT consumption can improve feed utilization efficiency by recovering a portion of voided nutrients and boosting nitrogen retention from additional feed by 7 to 13% [48,49,50]. In the present study, low-dietary protein diets were formulated to examine the compensation effect of BFT, in comparison to a commercial diet, whereas the recommended dietary protein level for *L. vannamei* is high, accounting for 35% [51]. Moreover, the development of BFT using a carbon source should be cost effective and not competitive to human consumption. Accordingly, in the present study, we use low-cost carbon sources, such as sugarcane bagasse (SB) and wheat flour (WF). According to the best available knowledge, little is known about how to improve shrimp performance by managing functional BFT by the addition of diverse carbon sources and the correct dietary protein amount. Therefore, the present study aims to investigate the effect of dietary protein levels (250, 300, and 350 g protein kg^−1^) and two different carbon sources (SB and WF) to improve *L. vannamei* production, feed utilization, and water quality.

## 2. Materials and Methods

### 2.1. Whiteleg Shrimp Juveniles

A 75-day feeding experiment was conducted at the National Institute of Oceanography and Fisheries (NIOF), Suez Branch. Juveniles of *L. vannamei* (average initial weight, 0.23 ± 0.04 g) were obtained from a commercial shrimp hatchery in the Al-Deba triangle, Damietta, Egypt. Shrimp were acclimatized in an indoor glass aquarium for 14 days before the feeding trial began at a temperature of 29.1 ± 0.2 °C, pH of 8 ± 0.01, and salinity of 20 ± 0.1 g L^−1^; during this period, they were fed twice daily with a control diet containing 450 protein (C_450_).

### 2.2. Experimental Design and Set-Up

The current experiment was conducted in glass aquaria (30 × 40 × 60 cm) filled with 80 L of filtered (50 µm) seawater. To adjust the salinity at 20 g L^−1^, the seawater was diluted with de-chloride tap water. At the beginning of the trial, juveniles of whiteleg shrimp were carefully selected from the stock tanks and directly distributed into 21 aquaria (3 replicates per treatment) with an initial stocking density of 13 shrimp/aquarium. Three levels of dietary protein (250, 300, and 350 g protein kg^−1^ diet) were used under two different carbon sources (SB and WF) to produce six treatments (SB_250,_ WF_250_, SB_300_, WF_300,_ SB_350_, and WF_350_, respectively), compared to the control diet without carbon addition and fed a practical diet (450 protein; C_450_). The formulated experimental diets were isolipidic (≈87.7 g kg^−1^) and isocaloric (gross energy ≈ 19.27 MJ kg^−1^ diet). The formula and proximate chemical composition of the experimental diets are presented in Table 1.

The experimental diets were prepared in the laboratory. Briefly, raw materials were crushed in an electric grain grinder (HR-40B Wuyi, Zhejiang, China) and sieved and weighed on a digital scale according to the formula (Table 1). All the ingredients of each diet were mixed with an electric blender until they formed a homogenous mixture. During the mixing process, 300 mL of warm water per kilogram of raw material was added and mixed for 15 min. This mixture was formed into pellets by extrusion through a pelleting machine (MG-2000 Tornado, Cairo, Egypt) with a 1 mm die. Finally, the pellets were dried in an oven at 55–60 °C (FD 260 Binder, Tuttlingen, Germany), then stored in plastic bags at 4 °C. The proximate compositions of experimented diets were analyzed, as described by AOAC [52].

Shrimp were fed with experimental diets at 10% of live body weight, which decreased gradually according to the increase in body weight by 3% at the end of the experiment. The daily-feed ratios were divided into three equal amounts given three times at 8:00, 14:00, and 20:00 h to all aquaria. During the experiment, all aquaria were supported with 5 cm air stones, which were fitted to the air compressor (220 W). Aeration was provided for 24 h throughout the experiment for ensuring better flocculation. Shrimp were held under a lighting regime (12:12 h, light:dark). All aquaria were covered with a plastic hab net to prevent jumping shrimp.

To initiate the development of BFT in the aquaria, 100 mL of BFT from an old shrimp tank was used to inculcate the experimental aquaria. The C/N ratio was 16:1 [48,53] after adding external carbon sources in the BFT treatments as SB (SB_250_, SB_300,_ and SB_350_) and WF (WF_250_, WF_300,_ and WF_350_). To convert 1 g of TAN into bacterial biomass, an average of 10 g of carbon is required [9]. In the control treatment (without carbon sources), water was exchanged two times per week. Meanwhile, BFT treatments were maintained without any water exchange, except for the addition of dechlorinated fresh water to compensate for evaporation loss and keep the salinity at 20 g L^−1^.

### 2.3. Water Quality Analysis

Water temperature, salinity, pH, TAN, and total suspended solids (TSSs) were all measured during the experiment to keep water quality within the shrimp optimum range. Water temperature was recorded daily at 13:00 h using a mercury thermometer suspended at a 30 cm depth. The salinity and pH were measured daily between 9:00 and 10:00 h by using a refractometer and pH meter (Orion pH meter, Abilene, TX, USA), respectively. From each tank, a 500 mL water sample was collected weekly and divided into two portions; the first was used to spectrophotometrically analyze TAN, NO_2_-N, and NO_3_-N, as described by Parsons [54]. Under vacuum pressure, the remaining portion of water sample was filtered using pre-dried and pre-weighed Whatman GF/C filter paper, then dried in an oven at 105 °C, and weighed to 0.01 mg. The weight difference was determined and a TSS estimate was obtained [55].

### 2.4. Carbon Sources and Biofloc Analysis

The locally available sources of SB and WF were selected as carbon sources for BFT treatments. SB was purchased as a by-product from a local vegetable market, then dried in an oven at 60 °C until totally dry, powdered, sieved at 35 μm, and then kept until further use [15]. WF powder was purchased from the local store: Suez Government, Egypt. The biochemical compositions (% dry-weight basis) of both SB and WF were determined following the standard methods of AOAC [52], while the organic carbon content (%) in SB and WF was determined as in Jackson [56]. However, the biochemical compositions and organic carbon content of both carbon sources (SB and WF) are presented in Table 2.

The volume of the BFT was measured biweekly using the Imhoff cone by registering the volume of the BFT in 1000 mL of tank water after 15–20 min of sedimentation, according to Avnimelech and Kochba [55]. The proximate composition of the BFT was determined following the standard methods of AOAC [52]. Briefly, the moisture content was determined by oven drying to a constant weight at 105 °C. Crude protein was estimated using Kjeldahl techniques and the factor of N × 6.25 [57] after acid digestion using an auto Kjeldahl system (Kelplus, DXVA, Pelican Equipments, Chennai, India). Using a Soxtec system (Socs plus, SCS-6, Pelican Equipment, Chennai, India), crude lipid was determined by the ether extraction method [58]. In a muffle furnace, ash content was determined by incineration at 600 °C for 6 h. Fibertec (Foss Tecator 2022, Sweden) was used to determine crude fiber utilizing sequential digestion with H_2_SO_4_ and NaOH.

### 2.5. Total Heterotrophic Bacteria

The total heterotrophic bacteria (THB) count was determined at the end of the experiment. From the center of the tanks, all samples were collected in a sterilized polypropylene bottle. Following that, a 10-fold serial dilution using sterilized distilled water was prepared. A total of 0.1 mL of appropriate dilutions was applied in duplicate over the tryptone soya agar containing 1.0% *w*/*v* NaCl. All plates were incubated at 37 °C for 24–48 h and colonies in the range of 30–300 counted and expressed as a bacterial colony-forming unit (cfu) according to [59,60].

### 2.6. Growth Performance and Feed Utilization Parameters

At the end of the experiment, shrimps were collected after draining the tanks’ water. Shrimp weight gain (WG), specific growth rate (SGR), feed conversion ratio (FCR), the protein efficiency ratio (PER), and survival rate (SR %) were calculated using the following equations:

Weight gain (WG) = Final body weight (g) − Initial body weight (g)(1)

Specific growth rate (SGR %) = [(Log final weight − log initial weight) × 100]/rearing period in days(2)

Feed conversion ratio (FCR) = Dry weight of feed consumed (g)/weight gain (g)(3)

Protein efficiency ratio (PER) = Weight gain (g)/Protein fed (g)(4)

Survival rate (SR % day^−1^) = (Final number of shrimp juveniles/Initial number of shrimp juveniles) × 100 (5)

### 2.7. Statistical Analysis

The presented data are means ± SD. All data were tested for homogeneity by Levene’s test, and the normal distribution was also checked using the Shapiro–Wilk’s test and all data met the parametric test requirements. All measured variables were performed using the SPSS ver. 16 to analyze the statistical difference one-way ANOVA, followed by the Duncan test [61]. In addition, two-way ANOVA was used to determine the effects of the protein level, carbon source, and their interaction [62]. Significance was set as *p* < 0.05.

## 3. Results

### 3.1. Water Quality

**Figure 1:**
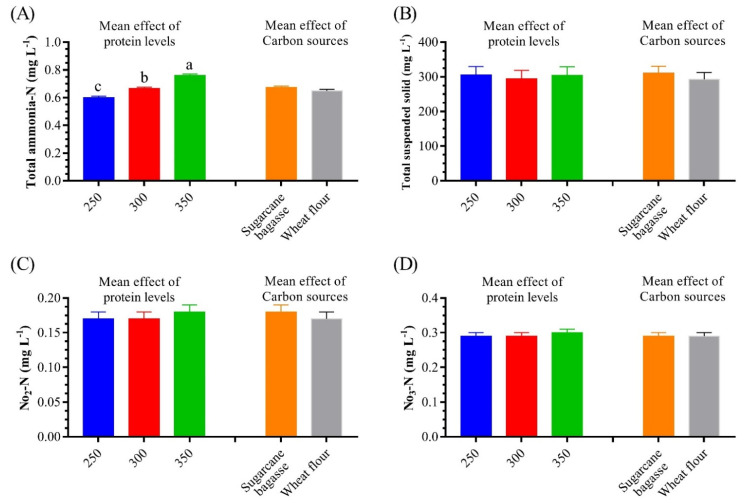
Mean effect of dietary protein levels and carbon sources on some water quality parameters of whiteleg shrimp *L. vannamei* juveniles reared in water. (**A**) Total ammonia nitrogen, (**B**) total suspended solids, (**C**) nitrate, and (**D**) nitrite. Different letters indicate significantly different values (*p* < 0.05).

All water quality conditions were at the permissible range for cultured *L. vannamei*, according to Mohanty et al. [63]. The different water parameters’ mean of temperature, pH, salinity, TTS, TAN, NO_2_-N, and NO_3_-N were 27.4 ± 0.17 °C, 7.55 ± 0.04, 20.6 ± 0.10 g L^−1^, 314.2 mg L^−1^, 0.674 ± 0.03 mg L^−1^, 0.181 ± 0.01 mg L^−1^, and 0.299 ± 0.003 mg L^−1^, respectively. Figure 1 does not show any significant effects of protein levels and carbon sources on the water quality of the BFT systems. Meanwhile, the main effect of increasing dietary protein levels shows a significant increase in TAN.

### 3.2. Biofloc Volume and Nutritional Values

**Figure 2:**
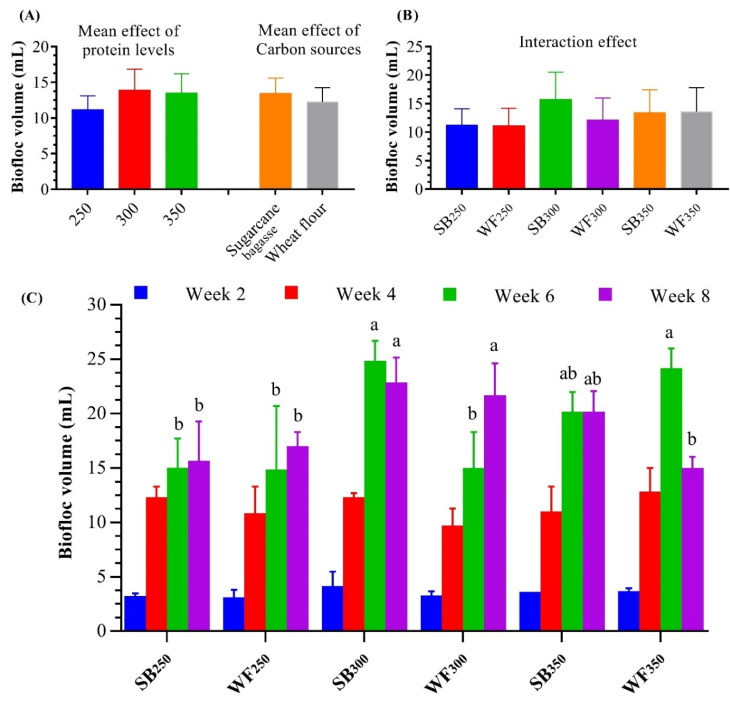
Effect of dietary protein levels and carbon sources on biofloc volume of whiteleg shrimp, *L. vannamei,* juveniles reared in water. (**A**) Mean effects of protein levels and carbon source, (**B**) interaction effect of protein levels and carbon source, and (**C**) biweekly in biofloc development. Presented data are means ± SD (*n* = 3). Different letters in the same week indicate significantly different values (*p* < 0.05).

Figure 2 shows the BFT volume (BFV) produced biweekly for 8 weeks (W2, W4, W6, and W8) in water tanks with BFT treatments. In W2 and W4, no significant differences (*p* < 0.05) in BFV were obtained for any BFT treatments. In W6 and W8, the highest significant (*p* < 0.05) BFV was obtained for SB_300_, followed by SB_350_, WF_350_, and WF_300_, while the lowest significant (*p* < 0.05) BFV was obtained for WF_250_ and SB_250_ (Figure 2), respectively. In general, among all BFT treatments, the highest BFV average of all weeks was reported at SB_300_ (9.63 mg L^−1^), followed by WF_350_ (8.42 mg L^−1^), SB_350_ (8.02 mg L^−1^), WF_300_ (7.81 mg L^−1^), WF_250_ (6.12 mg L^−1^), and SB_250_ (5.73 mg L^−1^). The two-way ANOVA study showed an increase in BFT volume with increasing dietary protein levels and SB as a carbon source.

**Figure 3:**
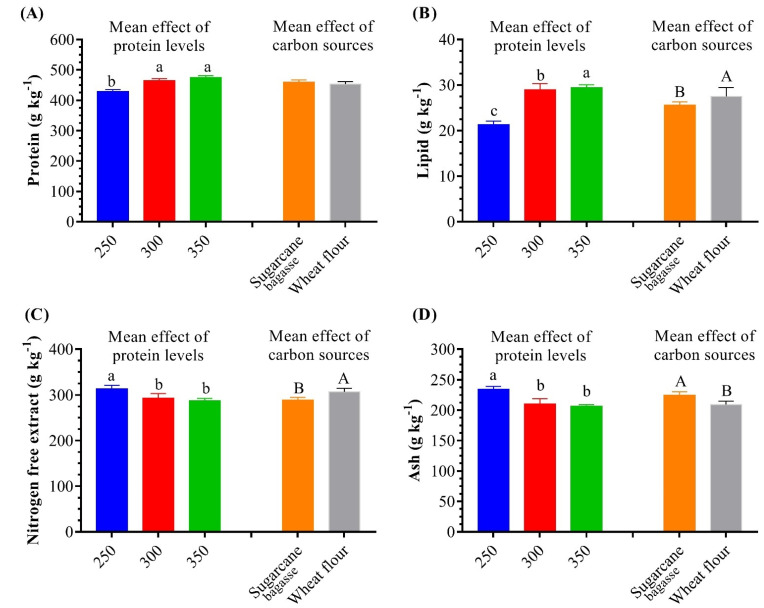
Mean effect of dietary protein levels and carbon sources on biofloc proximate chemical composition of whiteleg shrimp, *L. vannamei*, juveniles reared in water. (**A**) Protein content, (**B**) lipid content, (**C**) nitrogen-free-extract content, and (**D**) ash content. Presented data are means ± SD (*n* = 3). Different letters indicate significantly different values (*p* < 0.05).

Figure 3 and Table 3 show the biochemical compositions of different BFT treatments, depending on both protein levels and different carbon sources. In all BFT treatments (% dry-weight basis), crude protein content ranged from 42.63% to 47.98%, lipid content ranged from 1.98% to 3.2%, and nitrogen-free extract content ranged from 27.48 to 32.49%, while ash content ranged from 19.18 to 24.13%. The highest (*p* < 0.05) protein values were recorded for groups fed diets with protein contents of 300 g protein kg^−1^ (SB_300_ and WF_300_) and 350 (SB_350_ and WF_350_), compared to 250 (SB_250_ and WF_250_). The crude lipid content was significantly (*p* < 0.05) higher in WF_300_, SB_350_, and WF_350_ than SB_300_ and SB_250_. The nitrogen-free extract was higher (*p* < 0.05) in groups WF_250_, WF_250_, and WF_300_ than the other BFT treatments. The ash content significantly decreased with increased dietary protein levels, and using WF than SB.

The results show that increasing dietary protein levels significantly increases BFT protein and lipid contents, and decreases nitrogen-free extract and ash contents in the produced BFT. In addition, using WF as a carbon source significantly increased lipid and nitrogen-free extract contents. The interaction of dietary protein levels and different carbon sources was significant for lipids and ash and nonsignificant for protein content and nitrogen-free extract.

### 3.3. Microbial Community/Total Bacteria Count

**Figure 4:**
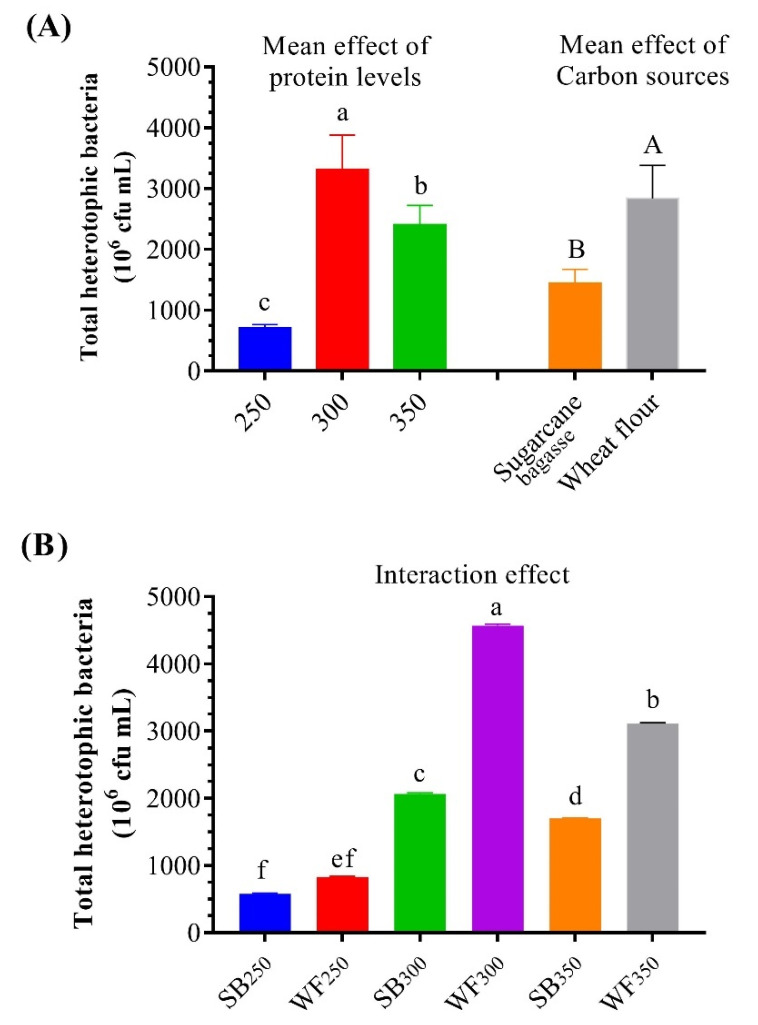
Effect of dietary protein levels and carbon sources on the total heterotrophic bacterial count in experimental tanks’ water. Presented data are means ± SD (*n* = 3). (**A**) Mean effect of protein levels and carbon source, and (**B**) interaction effect. Column bearing different letters is significantly different (*p* < 0.05).

The THB count was developed for overall treatments, especially BFT treatments. The addition of different carbon sources significantly (*p* < 0.05) affected the growth of the THB count, whereas WF significantly increased the THB count compared to SB. The highest significant THB count was recorded for 300 g protein kg^−1^. The mean effect of the protein levels showed a significant increase in the THB count with 300 g protein kg^−1^. In addition, the interaction effect of dietary protein levels and carbon sources revealed that the THB count was significantly higher (*p* < 0.05) in WF_300_ and WF_350_ than in the other treatments (Figure 4).

### 3.4. Growth Performance

**Figure 5:**
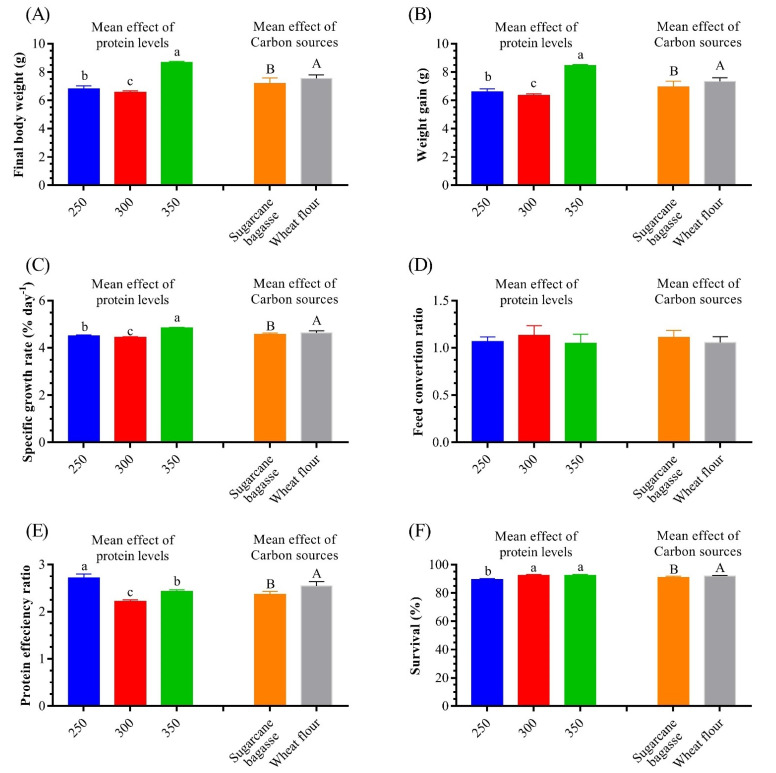
Mean effect of dietary protein levels and carbon sources on growth performance and feed utilization of whiteleg shrimp, *L. vannamei,* juveniles. (**A**) Final weight, (**B**) weight gain, (**C**) specific growth rate, (**D**) feed conversion ratio, (**E**) protein efficiency ratio, and (**F**) survival. Presented data are means ± SD (*n* = 3). Different letters indicate significantly different values (*p* < 0.05).

The results of the growth performance are presented in Figure 5 and Table 4. The main effects of increasing dietary protein levels showed a significant increase in FW, WG, SGR, and survival with the highest dietary protein levels. In addition, the shrimp reared in WF-based BFT systems had a higher growth performance than the shrimp reared in BS-based BFT. The interaction of both variables showed that there was a significant difference in the FBW, WG, PER, and SR% among the BFT treatments and the control. FBW and WG were significantly higher in SB_350_. However, there was no significant difference in FCR in all treatments_._ PER was significantly higher (*p* < 0.05) in WF_250_ than in the other treatments. The highest significant (*p* < 0.05) SR% was recorded at C_450_ (control diet), SB_300_, and WF_350_ (93.94%, 93.97%, and 93.97%, respectively), as presented in Table 4. The two-way ANOVA revealed high significant effects of both dietary protein levels and carbon sources in the BFT systems on growth performance, whereas the shrimp that were fed the highest protein levels had the highest FBW, WG, and SR.

## 4. Discussion

The importance of BFT technology for shrimp culture has been reported, particularly in intense culture systems with negligible water exchange [10,12,30,36,53,64]. In the current study, the BFT was promoted through the appropriate addition of different carbon sources. The BFT volume and TSS, in addition to the other water quality conditions, were gradually increased and kept within optimum ranges. The formation and development of BFT in shrimp water depended on the assimilation of dissolved nitrogenous substances from uneaten feed and shrimp excretions by THB [55,65]. The optimum C/N ratio (16:1) promoted the growth of THB in the water column and improved the BFT composition, in comparison to the control diet [48,53]. The increased bacterial load resulted in a faster degradation of organic wastes, releasing inorganic nutrients, which stimulated the development of protein-rich new bacterial cells [53]. As a result of the development of the heterotrophic community, single-cell bacterial protein was formed, which served as a protein source for shrimp, leading to a higher growth rate. In the present study, the higher THB load/count developed in BFT treatments increased the growth of *L. vannamei* compared to the control treatment. This could be due to the assimilation of the shrimp to the formed BFT as additional supplemental food [30,53]. In addition, Heo et al. [66] suggested that the *Lactobacillus* strain that is abundant in BFT is a good candidate feed additive to improve fish growth and enhance immune responses. Bacteriocins produced by lactic acid bacteria have gained a lot of interest because they are used as natural preservatives in food [67].

The current results show that the THB count increases gradually at the end of the experiment, which agrees with the study conducted by de Paiva Maia et al. [68], who found that the THB count increased in BFT treatments in the 10th and 12th weeks, indicating that THB might effectively assimilate the TAN to generate bacterial protein and new cells if an appropriate carbon source was used [9,59,60]. In the current study, the additions of different carbon sources of SB and WF stimulated THB growth in the aquarium water. The increase in THB could improve water quality via immobilizing inorganic nitrogen [60,69], which is the most common waste material in aquaculture, and by converting this waste into microbial protein [9], it could be used as a supplemental meal for shrimp and improve digestive enzyme activity [11].

The nutritional composition of BFT has been observed to be influenced by the carbon source [22]. However, there is a scarcity of information on the effect of dietary protein levels and the addition of carbon sources in penaeid shrimp farming on growth performance and immunological response. Although the involvement of BFT in stimulating shrimp growth is mainly unknown, it could produce a variety of effects. The whiteleg shrimp were found to be able to utilize and digest BFT in situ, which was available 24 h a day in the rearing water [53]. As a result, BFT could improve shrimp growth rates in culture systems as an essential and extra natural food supply [16,18]. It not only provided vital macronutrients (protein, lipids, and carbohydrates), but it also met the shrimp’s mineral and vitamin needs [13,70,71].

From the results of the present study, it can be observed that the BFT crude protein content is significantly high in the treatments of SB_300_, WF_300_, SB_350,_ and WF_350_, which means that the BFT protein content increases with increasing the dietary protein level. However, it could be noted that reducing the dietary protein level from 350 to 250 g protein kg^−1^ could not affect the BFT protein composition [14]. In a proximate analysis, the used carbon source, TSS level, salinity, stocking density, light intensity, phytoplankton, zooplankton, and bacterial populations influenced the nutritional characteristics of BFT [72]. In addition, Xu and Pan [13] found that the proximate chemical analysis of the BFT produced in *L. vannamei* water showed that BFT had a crude protein content comparable with their practical diet, with a range of 25–320 g protein kg^−1^. Our results agree with Rajkumar et al. [70] who found that the protein, lipid, and fiber contents of BFT produced in BFT with WF in *L. vannamei* tanks were significantly higher than those of the control. The high protein content was observed in the BFT produced in the BFT with WF followed by BFT with sugarcane molasses. BFT with more than 25% crude protein, 4% fiber, and 7% ash can be regarded as acceptable for aquatic animal nutrition, particularly for herbivorous/omnivorous fish and shrimp species [73].

Generally, the protein level in the shrimp diet is the most influential factor in shrimp culture. In this study, the shrimp fed on SB_350_ and WF_350_ g protein kg^−1^ had a better growth performance, compared to the shrimp fed on C_450_. This means that shrimp fed lower levels of protein with BFT could compensate for the higher protein diet (control diet). In agreement with the present study, the growth performance of *L. vannamei* reared in BFT systems was not affected by using different dietary protein levels, such as 30 and 450 g protein kg^−1^ for post-larvae in ponds [74], 250–350 and 350–400 g protein kg^−1^ for juveniles in tanks [13,23], and 350–400 g protein kg^−1^ for juveniles in raceways [75]. On the other hand, several studies found that increasing the protein content in food improves shrimp performance over a lower range of 210–310 g protein kg^−1^ for juveniles in tanks and 250–350 g protein kg^−1^ for adults [14,45].

The addition of the appropriate amount of carbon aided *L. vannamei* growth and survival. This could be due to the combined impact of better water quality and higher bacterial and zooplankton concentrations [76]. Several studies have indicated that carbon-source addition has been linked to the formation and accumulation of BFT [77,78], which could provide an essential food source or a safe shelter for zooplankton, boosting fish and shrimp growth, in addition to their environmental role in aquatic ecosystems [79,80]. Furthermore, zooplankton has been shown to be a supplemental food supply for *L. vannamei*, possibly increasing the efficiency of microbial protein conversion to animal protein [81,82].

In the current study, improved values of PE were observed in the BFT treatments. PER values were highly significant in SB_250_ and WF_250_ than in the other treatments. When there is a high abundance of BFT in the culture system, feeding shrimp a high-protein diet may be unnecessary and uneconomical. In other words, a low level of dietary protein might be compensated for by consuming BFT, allowing for the dietary protein content to be reduced. The BFT influence on protein feeding could have contributed to the shrimps’ good development in the BFT treatments. In accordance, *L. vannamei* and *P. monodon* juveniles had higher growth rates in the BFT-based system than others reared in clear water [16,18].

In the current study, the final weight, WG, and SGR of *L. vannamei* in the BFT treatments fed diets containing 250, 300, and 400 g protein kg^−1^ were markedly higher than those obtained in the control groups that were fed diets containing 450 g protein kg^−1^. Accordingly, rearing shrimps in the BFT system could compensate for the reduction in dietary protein. More significantly, by decreasing diet protein, the fishmeal level decreased; a low-protein diet is more cost-effective and environmentally beneficial [5]. The reduction in dietary protein levels without affecting shrimp growth has been reported in several studies, whereas microbial proteins could provided an alternative source of protein for shrimp in the BFT systems [48,83]. In a similar study, Decamp et al. [84] reported no significant changes in the growth performance of *L. vannamei* grown in unfiltered pond water and fed either a 25 or 35 percent CP diet. In addition, the SGR of *P. monodon* fed on a 250 and 400 g protein kg^−1^ diet did not significantly differ when reared in a BFT system, even under intensive culture [48]. The better growth and survival rates of *L.* *vannamei* may be due to the improved water quality as a result of flocculation in a zero-water exchange system, which is caused by adding organic carbon sources in a correct C/N ratio to the system [85]. In addition, Yun et al. [14] and Browdy et al. [74] found that the growth of juvenile whiteleg shrimp *L. vannamei* was not affected by decreasing dietary protein from 350 to 300 or lower. Moreover, FCR could be maintained in shrimp reared in a BFT-based system, even with reducing dietary protein by around 10% [23].

## 5. Conclusions

The results of the present study reveal that different carbon sources and protein levels could effectively influence the nutritional value of biofloc. The current study suggested that, by rearing whiteleg shrimp, *Litopenaeus vannamei*, on a biofloc system, dietary protein levels may be reduced from 450 to 350 g protein kg^−1^ diet with improving the growth performance and feed utilization of shrimp using biofloc technology. In addition, the applied biofloc improved the growth performance and feed utilization of the shrimp, probably by providing a supplemental food source with appropriate protein and lipid contents. The total heterotrophic bacterial count in WF_300_ and WF_350_ g protein kg^−1^ (using wheat flour as a carbon source) was significantly higher than in the other treatments. The 350 g protein kg^−1^ provided the best performance indices, demonstrating the greater economic viability of shrimp.

## Figures and Tables

**Table 1:** Ingredients and proximate composition of the experimental diets (g kg^−1^).

Ingredients (g kg^−1^)	Experimental Diets (g Protein kg^−1^ Diet)			Control Diet (450 g Protein kg^−1^ Diet)
	250	300	350	
Fish meal	90	270	270	450
Soybean meal	330	230	330	330
Yellow corn	280	250	205	90
Wheat bran	195	155	100	45
Fish oil	60	50	50	40
Cholesterol	5	5	5	5
Di-calcium phosphate	20	20	20	20
Vitamin and minerals ^1^	20	20	20	20
Proximate analysis (g kg^−1^)				
Dry matter	893.5	899.6	901.8	906
Crude protein	256.2	319.6	352.1	451.7
Crude lipid	71.8	92.6	89.3	81.2
Ash content	78.1	81.2	90.1	112.3
Crude fiber	49.8	39.3	39.8	33.8
Nitrogen-free extract ^2^	544.1	467.3	428.7	321
Gross energy (MJ kg^−1^ diet) ^3^	18.70	19.22	19.20	19.38

^1^ Vitamin premix (mg or IU kg^−1^ diet): vitamin A, 6000 IU; vitamin D3, 2000 IU; ascorbic acid, 200 mg; vitamin E, 50 mg; menadione, 5 mg; thiamine, 15 mg; riboflavin, 15 mg; nicotinic acid, 30 mg; pantothenic acid, 35 mg; pyridoxine HCl, 6 mg; cyanocobalamin, 0.03 mg; biotin, 0.2 mg; inositol, 200 mg; folic acid, 3 mg; iodine, 0.4 mg; cobalt, 0.1 mg; copper, 4 mg; iron, 150 mg; zinc, 80 mg; manganese, 20 mg; selenium, 0.1 mg; magnesium, 100 mg. ^2^ Nitrogen-free extract (%) = 100 − (crude protein + ether extract + crude fiber + ash). ^3^ Gross energy (MJ kg^−1^) = (crude protein × 23.6 + ether extract × 39.5 + Nitrogen-free extract × 17.2)/100.

**Table 2:** Biochemical compositions and organic carbon content (g kg^−1^) of used carbon sources.

Carbon Sources	Total Organic Carbon	Total Protein	Total Lipid	Nitrogen-Free Extract	Ash	Fiber
Sugarcane bagasse	395 ± 2.1	15.2 ± 0.1	15.3 ± 0.1	244 ± 0.3	76 ± 0.2	650 ± 2.1
Wheat flour	411 ± 1.1	122 ± 0.3	12.5 ± 0.2	812 ± 11	41.5 ± 0.2	13 ± 0.2

**Table 3:** Effect of dietary protein levels and carbon sources on nutritional value (g kg^−1^) of the biofloc produced in biofloc treatments.

Protein Levels (g kg^−1^)	Control (450) *	250		300		350		*p*-Value of Two-Way ANOVA		
Carbon Sources		SB	WF	SB	WF	SB	WF	Protein Levels	Carbon Source	Interaction
Protein	–	432.20 ± 13.00 ^b^	426.30 ± 18.50 ^c^	467.80 ± 17.70 ^a^	463.80 ± 15.20 ^a^	479.80 ± 15.60 ^a^	471.6 ± 16.30 ^a^	0.001	0.436	0.972
Lipid	–	23.00 ± 0.10 ^b^	19.80 ± 0.30 ^c^	26.00 ± 0.20 ^b^	32.00 ± 0.10 ^a^	28.00 ± 0.30 ^a^	31.00 ± 0.40 ^a^	<0.001	<0.001	<0.001
Nitrogen-free extract	–	303.50 ± 13.30 ^a^	324.90 ± 19.00 ^a^	274.80 ± 15.10 ^c^	312.40 ± 13.6 ^b^	290.30 ± 15.50 ^b^	285.50 ± 12.80 ^b^	0.028	0.026	0.090
Ash	–	241.3 ± 7.80 ^a^	229.00 ± 9.90 ^a^	231.40 ± 8.40 ^a^	191.80 ± 6.30 ^b^	201.90 ± 6.90 ^b^	211.90 ± 8.80 ^c^	<0.001	0.002	0.003

Means (mean ± SD) in the same row having superscript differ significantly (*p* < 0.05); two-way ANOVA significant at *p* < 0.05. * The data of residues collected from control tanks are excluded, however they do not represent any importance to the study. Means with different letters is significantly different (*p* < 0.05).

**Table 4:** Growth indicators of whiteleg shrimp, *L. vannamei*, juveniles fed with different protein levels for the experimental period (75 days).

Protein Levels (g kg^−1^)	Control (450)	250		300		350		*p*-Value of Two-Way ANOVA		
Carbon Sources		SB	WF	SB	WF	SB	WF	Protein Levels	Carbon Source	Interaction
FBW	6.83 ± 0.04 ^d^	6.35 ± 0.02 ^e^	7.31 ± 0.01 ^c^	6.37 ± 0.07 ^e^	6.80 ± 0.02 ^d^	8.83 ± 0.03 ^a^	8.55 ± 0.07 ^b^	0.001	0.001	0.001
WG	6.60 ± 0.04 ^d^	6.12 ± 0.02 ^e^	7.08 ± 0.01 ^c^	6.14 ± 0.07 ^e^	6.57 ± 0.02 ^d^	8.60 ± 0.03 ^a^	8.32 ± 0.07 ^b^	0.001	0.001	0.001
SGR	4.52 ± 0.01	4.43 ± 0.01	4.61 ± 0.01	4.43 ± 0.02	4.52 ± 0.01	4.86 ± 0.01	4.82 ± 0.01	0.001	0.001	0.10
PER	1.52 ± 0.01 ^e^	2.54 ± 0.01 ^b^	2.92 ± 0.01 ^a^	2.12 ± 0.01 ^d^	2.26 ± 0.01 ^c^	2.52 ± 0.01 ^b,c^	2.44 ± 0.01 ^b^	0.001	0.001	0.001
FCR	1.30 ± 0.36	1.09 ± 0.11	0.97 ± 0.09	1.25 ± 0.34	1.03 ± 0.09	0.93 ± 0.08	1.17 ± 0.32	0.74	0.55	0.11
SR	93.94 ± 3.32 ^a^	87.88 ± 5.92 ^c^	90.91 ± 4.57 ^b^	93.97 ± 5.74 ^a^	90.91 ± 6.55 ^b^	90.91 ± 4.57 ^b^	93.97 ± 3.45 ^a^	0.001	0.001	0.001

Means (mean ± SD) in the same row have superscripts that differ significantly (*p* < 0.05); two-way ANOVA significant at *p* < 0.05. FBW: final body weight (g), WG: weight gain (g), SGR: specific growth rate (% day^−1^), FCR: feed conversion ratio, PER: protein efficiency ratio, and SR: survival rate (%). Means with different letters is significantly different (*p* < 0.05).

## Data Availability

Not applicable.
